# Impact of stroke on respiratory function and amyloid-β pathology in Tg-2576 mice

**DOI:** 10.1177/13872877261476022

**Published:** 2026-08-17

**Authors:** Yu-Xing Zhang, Ahmad El Hamamy, Zahid Iqbal, Arya Ranjan, Destiny Sumani, Karienn A. De Souza, Juneyoung Lee, Sean Marrelli, Louise D. McCullough, Jun Li

**Affiliations:** 1 Department of Neurology, McGovern Medical School, University of Texas Health Science Center at Houston, Houston, TX, USA; 2 Department of Neuroscience and Experimental Therapeutics, Texas A&M Naresh K. Vashisht College of Medicine, Bryan, Bryan, TX, USA

**Keywords:** Alzheimer's disease, breathing dysfunction, cognition impairment, retrotrapezoid nucleus

## Abstract

**Background:**

Stroke is a well-established risk factor for dementia, and many patients with Alzheimer's disease exhibit mixed neuropathology that includes both ischemic injury and amyloid-β (Aβ) accumulation. Breathing disturbances, such as apnea, have also been linked to cognitive dysfunction and accelerated dementia progression.

**Objective:**

We hypothesized that stroke aggravates respiratory dysfunction and cognitive impairment in Tg-2576 mice.

**Methods:**

Female Tg-2576 mice (13–17 months old) underwent permanent distal middle cerebral artery occlusion (pd-MCAO), with age- and sex-matched wild-type and sham-operated controls. Cognitive performance was assessed using the Barnes maze. Respiratory parameters were quantified by whole-body plethysmography. Immunofluorescence was performed to measure Aβ deposition in hippocampus and cortex, astrocyte reactivity in retrotrapezoid nucleus (RTN) using GFAP, and LYVE1 in deep cervical lymph nodes (dCLNs). Aβ levels in cerebrospinal fluid were also assessed as a readout related to clearance-associated changes.

**Results:**

Compared with wild-type controls, Tg-2576 mice exhibited increased apnea frequency and impaired cognitive performance. Following pd-MCAO, Tg-2576 mice showed a further increase in apnea events and prolonged escape latencies in the Barnes maze. Stroke was also associated with enhanced astrocyte reactivity in the RTN, increased Aβ deposition in the hippocampus and cortex, and reduced Aβ levels in cerebrospinal fluid, along with decreased LYVE1-positive lymphatic area in dCLNs, suggesting compromised glymphatic–lymphatic clearance.

**Conclusions:**

Collectively, these findings indicate that stroke worsens respiratory dysfunction, impairs Aβ clearance pathways, and accelerates cognitive decline in Tg-2576 mice. Targeting post-stroke respiratory abnormalities may represent a therapeutic avenue to mitigate dementia-related comorbidity after ischemic injury.

## Introduction

The growing aging population has increased the incidence and prevalence of neurological diseases, with amyloid-associated disorders such as Alzheimer's disease (AD), cerebral amyloid angiopathy (CAA) and ischemic stroke becoming an escalating public health concern.^1–3^ AD is characterized by progressive amyloid-β (Aβ) accumulation in the brain and is associated with an increased risk of cerebrovascular events as pathology advances.^4^ Aβ-associated vascular injury increases susceptibility to stroke and can exacerbate ischemic brain damage by compromising cerebrovascular integrity and cerebral perfusion.^5^ Conversely, ischemic stroke may accelerate Aβ deposition and downstream pathology, thereby exacerbating cognitive decline.^6,7^ Specifically, AD is a chronic neurodegenerative disorder involving age-dependent Aβ aggregation and plaque formation, with prominent involvement of cognition-related regions such as the cortex and hippocampus, which are essential for memory and learning.^8^ This progressive Aβ burden contributes to synaptic dysfunction, impaired neurovascular coupling, and chronic hypoperfusion, promoting cerebral infarction, microvascular injury, and vascular-associated cognitive impairment.^5,9^ Over time, sustained Aβ accumulation amplifies neuroinflammation and compromises blood–brain barrier (BBB) integrity, further aggravating neuronal injury and functional deterioration.^10^ Although previous studies have largely emphasized parenchymal plaque pathology in AD, emerging evidence underscores the need to better define its ischemic and neurovascular components.

AD is an important contributing factor in stroke occurrence. Over half of individuals with AD experience at least one stroke episode, and clinical outcomes are typically more severe, with mortality rates higher than those observed in patients without AD.^11,12^ Beyond neurodegeneration, AD is accompanied by prominent cerebrovascular dysfunction, including impaired neurovascular coupling, endothelial dysfunction, and reduced cerebral perfusion, all of which increase vulnerability to cerebral ischemia and worsen post-stroke outcomes.^13^ Individuals with AD may be more susceptible to ischemic stroke, as AD is associated with chronic cerebral hypoperfusion, impaired cerebrovascular reactivity, and reduced vascular/BBB clearance of amyloid-β, factors that may together limit vascular reserve and adaptive responses under ischemic stress.^14,15^ Furthermore, AD-associated small-vessel pathology and white matter degeneration are linked to silent infarcts and progressive cognitive decline, thereby amplifying the long-term neurological burden after cerebrovascular events.^16,17^ The presence of AD can also complicate ischemic stroke recovery by limiting vascular and synaptic repair, exacerbating neuroinflammation, and increasing the likelihood of recurrent cerebrovascular events. In addition, AD has been increasingly linked to respiratory dysfunctions, especially sleep apnea, which involves repeated interruptions in breathing during sleep.^18^ This condition not only raises cardiovascular risks but also aggravates brain hypoxia, neuroinflammation, and cognitive impairment, adding to the complexity of managing these disorders.^19^

Our prior work showed that AD model mice exhibit more frequent apnea events and a slower respiratory rate, changes that are attributable, at least in part, to heightened TGF-βR II and reactive gliosis within the retrotrapezoid nucleus (RTN) of the brainstem.^20^ The RTN, located in the ventral medulla, contains a population of specialized glutamatergic neurons, and serves as a key hub for central CO_2_ chemosensitivity and respiratory rhythm control.^21,22^ In rodents, RTN circuitry contributes to blood-gas homeostasis by engaging feedback control that promotes O_2_ uptake and CO_2_ removal.^23^ Importantly, our previous findings further suggest that RTN gliosis is causally involved in AD-related breathing abnormalities and may accelerate cognitive deterioration.^20^ In contrast, how stroke modifies RTN pathology and respiratory dysfunction in AD remains unclear. Notably, impaired breathing has been linked to cognitive decline and disrupted Aβ clearance, in part through dysfunction of the glymphatic system, a brain-wide fluid transport pathway that removes metabolic waste.^24,25^ Following stroke, glymphatic transport is substantially compromised, with reduced interstitial solute clearance and impaired cerebrospinal fluid (CSF) flow, which can facilitate the accumulation of neurotoxic proteins such as Aβ.^6,26^ Together, these observations highlight the potential importance of preserving brainstem respiratory control, particularly within the RTN, to support glymphatic clearance and possibly slow AD progression.

These observations are highly relevant because impaired glymphatic transport is expected to promote the retention of neurotoxic solutes, including Aβ.^27^ Moreover, respiratory dysfunction, particularly recurrent apneas, may further suppress glymphatic clearance by disrupting sleep architecture and altering CO_2_/O_2_ homeostasis and vascular tone, thereby creating a feed-forward loop in which disordered breathing and vascular injury jointly accelerate amyloid-related pathology and functional decline.^28,29^ Accordingly, systematic assessment of glymphatic drainage and respiratory physiology in AD stroke models is needed to determine whether ischemia precipitates a disproportionate decline in clearance capacity in the context of parenchymal plaque pathology, to clarify how post-stroke respiratory dysregulation contribute to impaired brain Aβ removal, and to identify actionable mechanistic targets across brainstem respiratory control, and fluid-transport that may mitigate post-stroke cognitive deterioration and potentially slow AD progression. In this study, we hypothesized that stroke in AD mice would further exacerbate RTN astrogliosis, compromise respiratory function, impair glymphatic clearance, and increase cerebral Aβ accumulation, culminating in worsened cognitive deficits.

Because glymphatic transport depends on peri-/paravascular pathways, CSF–ISF exchange, and vascular pulsatility, the current amyloid pathologies in the Tg-2576 model, encompassing both parenchymal plaques and vascular amyloid, may render the brain uniquely vulnerable to ischemic injury. This multifaceted pathological landscape allows for a more comprehensive assessment of how combine AD-related burdens exacerbate glymphatic failure following stroke.^30^ Taken together, these considerations underscore the importance of investigating stroke–AD interactions with a complex pathological framework. Rather than viewing these as separate entities, the Tg2576 model allows us to examine how an acute ischemic insult “pushes” a glymphatic system that is already compromised by concurrent parenchymal plaques and CAA models. To this end, we utilized Tg-2576 mice, which develop progressive parenchymal Aβ deposition in the cortex and hippocampus without severe early neurodegeneration. This specific profile makes them an ideal model for interrogating the interaction between amyloid pathology and vascular injury in aged animals.

## Methods

### Animals

Given that female Tg2576 mice typically develop more extensive Aβ plaque deposition and exhibit more pronounced cognitive deficits than males, we focused this study on female mice to ensure a consistent pathological phenotype.^31,32^ Therefore, female Tg-2576 mice (13–17 months old) and age-matched wild-type (WT) littermates were used in this study. The Tg-2576 colony was maintained in-house by breeding male heterozygous mice with female WT mice to generate heterozygous experimental subjects and their respective WT littermate controls. Animals were housed in a climate-controlled vivarium in groups of five under a 12 h light/dark cycle with ad libitum access to food and water. For group allocation, each mouse within a cage was first assigned an identification number (1–5) by a laboratory member not involved in the experimental procedures, after which a random number generator was used to allocate mice to control or treatment conditions. Sample sizes were selected based on prior studies using similar models and endpoints to ensure sufficient statistical power for detecting differences in behavioral performance and relevant molecular alterations.

### Animal ethics declaration

All procedures complied with NIH guidelines for the care and use of laboratory animals and were conducted under protocols approved by the Institutional Animal Care and Use Committee (AWC-24-0126) of the University of Texas Health Science Center at Houston.

### Permanent distal middle cerebral artery occlusion (pd-MCAO) model

Permanent focal ischemia was generated by creating a right-sided distal MCA permanent lesion using thermal coagulation, following established procedures.^33^ Mice were first anesthetized with isoflurane delivered in air (4–5% for induction and 1.8–2% for maintenance). Throughout the operation, animals were kept normothermic on a warmed platform, with body temperature clamped at 37°C. To provide local analgesia, bupivacaine (0.25%, 1 mL/kg, subcutaneous) was injected before the incision. A small lateral scalp opening was then made in the temporal region (between the orbit and ear), and the temporalis muscle was carefully displaced to uncover the cranial surface over the distal MCA. After thinning the bone, a small burr hole was produced with a micro-drill to expose the artery under direct visualization. The distal MCA trunk was subsequently thermally sealed with a cauterizer, targeting the segment immediately proximal to the point where the vessel divides into anterior and posterior branches, thereby achieving permanent occlusion. Postoperatively, animals recovered with supplemental external warming for 2 h. Sham controls underwent identical anesthesia, exposure, and cranial opening, but no thermal sealing of the MCA was performed.

### Whole-body plethysmography

Respiratory patterns were measured using a mouse whole-body plethysmography setup as previously described.^34^ Plethysmography was performed in a dedicated testing room. Before recording, mice were transferred to the testing room and allowed to acclimate to the room environment for 30 min. Each mouse was then placed individually in the recording chamber and allowed to habituate to the chamber for 60 min. After chamber habituation, breathing was monitored for 20 min to obtain tidal volume, respiratory rate, and minute ventilation. Apneas were detected using a two-step threshold method. Based on published definitions,^34^ an apnea was classified as a pause in breathing lasting at least two times the average baseline breath interval. In line with prior work, interruptions shorter than 0.5 s were not counted. Apnea incidence was reported as apneas per minute for each animal.

### Barnes maze

Barnes maze testing was used to evaluate hippocampus-dependent spatial learning and memory as described previously.^20,35^ The apparatus comprised a brightly illuminated circular platform with 20 equidistant perimeter holes (5 cm in diameter). A dark, enclosed escape box was located beneath one designated target hole, providing a shelter from the aversive light/noise cues. An external noise stimulus was applied during each trial to motivate animals to locate the target rapidly, the noise was turned off immediately once the mouse entered the escape box or was gently guided into it. Mice completed acquisition training on four consecutive days, during which they were allowed up to 5 min to locate and enter the escape box, animals that did not reach the target within this period were gently directed to the correct hole. To minimize olfactory guidance, the surface was cleaned with 70% isopropyl alcohol between subjects. For each trial, the time to reach the target hole (latency) and the number of incorrect hole investigations (errors) were recorded. Memory retention was assessed on day 5 with a test trial.

### CSF collection

CSF was collected from the cisterna magna based on published procedures.^36^ Briefly, mice were deeply anesthetized with avertin and positioned in a stereotaxic apparatus with the head flexed to approximately 120° relative to the neck. The dorsal neck area was shaved and disinfected with iodine followed by 70% ethanol. A midline incision was made, and the overlying musculature at the occipital region was gently dissected using scissors and curved forceps to expose the dura covering the cisterna magna, a triangular space where one to two prominent basilar arteries are typically visible. The dura was then carefully perforated with the tip of a glass capillary; upon entry, CSF was allowed to flow passively into the collection tube. Samples were centrifuged at 2000 × g for 10 min to remove any blood contamination, and the supernatant CSF was immediately stored at −80 °C until analysis.

### Quantification of Aβ peptides enzyme-linked immunosorbent assay

To determine the amount of mouse Aβ_42_ peptides, the major component of amyloid plaques, in mouse CSF, commercial ELISA kits (Thermo Fisher Scientific, Waltham, MA, USA: KHB3442) were used according to the manufacturer's instructions. Briefly, 6 μL CSF was diluted in 54 μL sample dilution. Then, according to the manufacturer's instruction, the absorbance at 450 nm was measured using a Bio-Rad 680 microplate reader (Biotek, USA).

### Immunostaining

Following deep anesthesia with Avertin (300 mg/kg), mice were transcardially perfused first with ice-cold PBS and then with 4% PFA, as reported previously. Brains were dissected to collect cerebral hemispheres and brainstems, which were post-fixed in PFA for 24 h and then cryoprotected in 30% sucrose containing 0.02% sodium azide for 48 h. Tissues were sectioned at 25 μm using a sliding cryostat. Deep cervical lymph nodes (dCLNs) were isolated from the ventral neck region. Briefly, the sternocleidomastoid muscles were gently separated to expose the carotid sheath region, and the dCLNs located along the internal jugular vein and carotid artery were carefully dissected using fine forceps. The collected tissues were post-fixed, embedded, and sectioned for subsequent immunofluorescence analysis.

Mounted sections were washed in PBS and permeabilized for 15 min in PBS containing 0.1% Triton X-100, then blocked for 1 h in blocking buffer (3% donkey serum and 0.1% bovine serum albumin in 0.1% Triton X-100). For dCLN sections, paraffin-embedded slices were deparaffinized, rehydrated, subjected to antigen retrieval, and blocked according to standard procedures. Sections were incubated overnight at 4°C with primary antibodies against Aβ (1:300, Abcam, Cat# ab201060), Phox2B (1:20, Bio-Techne, Cat# AF4940), GFAP (1:150, Sigma-Aldrich, Cat# NE1015), and LYVE1 (1:200, CST, Cat# E3L3 V) diluted in blocking buffer. After washing with PBS, sections were incubated for 1 h at room temperature in the dark with species-appropriate secondary antibodies (1:500; Abcam: anti-goat Cat# ab150131, anti-rabbit Cat# ab150073, anti-mouse Cat# ab150115). Sections were mounted, counterstained with DAPI, and coverslipped. Negative controls were processed in parallel with omission of primary antibodies to assess non-specific secondary antibody binding. Images were acquired using a Leica Thunder Imaging System, and quantitative analyses were performed in Fiji (v2.15.1).

### Statistical analysis

All statistical analyses were performed using SPSS (Statistical Package for the Social Sciences, version 27.0). Data are expressed as the mean ± S.E.M., and p < 0.05 was considered statistically significant. All data were tested for normality using the Shapiro-Wilk test. Post hoc comparisons following a significant Kruskal–Wallis test were performed using Mann–Whitney U tests (non-normal distribution). For comparisons involving three or more groups, one-way analysis of variance (ANOVA) followed by the Bonferroni test were employed to analyze the data pertaining to multiple groups. All statistical charts were drawn using GraphPad Prism 10.0 and Photoshop 2021.

## Results

### Distal stroke exacerbates cognition impairment in mice with dementia

**Figure 1.**
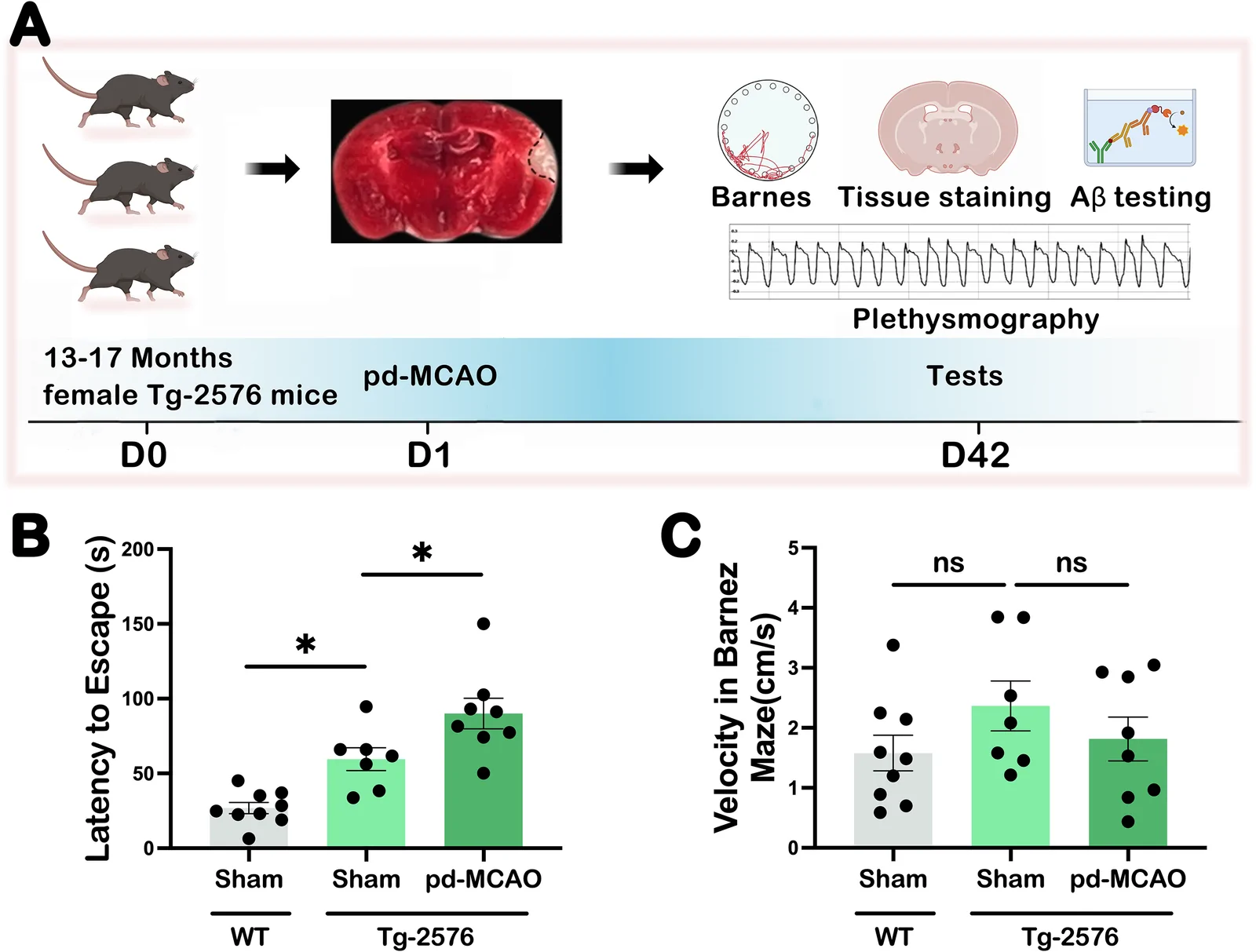
Distal stroke exacerbates cognitive impairment in Tg-2576 mice on day 42 post-stroke. (A) Schematic description of the experimental timeline for the pd-MCAO model, and behavioral tests, whole-body plethysmography test, and biological assessments on days 42 post-stroke. The brain slice image in the timeline is shown only as an illustrative graphic indicating the pd-MCAO procedure and was not used for penumbra or infarct quantification. (B) latency to escape chamber time in Barnes Maze test (F (2,21) = 19.106, p < 0.001). (C) mice Velocity in Barnes Maze (F (2,21) = 1.24, p = 0.31. *p < 0.05. ns: non-significance. n = 9 for WT, n = 7 for Tg-2576-Sham group, and n = 8 for Tg-2576-pd-MCAO group. The data are shown as the mean ± SEM. After performing the Shapiro-Wilk normality test to examine the normal distribution, One-way analysis of variance (ANOVA) was employed to analyze the data pertaining to multiple groups, subsequently, multiple comparisons were conducted using the Bonferroni test.

To evaluate whether distal MCAO influences cognitive performance in Tg-2576 mice, 13–17-months old female WT and Tg-2576 mice underwent either permanent distal-MCAO or Sham surgery (Figure 1A). Forty-two days after surgery, cognition function was accessed using the Barnes maze. As expected, AD mice (Tg-2576) with sham surgery (micro-drilling only, without MCA cauterization) exhibited impaired cognition, requiring more time to locate the escape chamber than WT controls (Tg: 59.5 ± 7.6 versus WT: 26.8 ± 3.7, *p* = 0.017, n = 7 and 9) in the Barnes Maze. Stroke further exacerbated memory deficits in Tg-2576 mice, as reflected by the increased latency relative to Tg-2576 Sham animals (MCAO: 90.05 ± 10.2 versus 59.5 ± 7.6, *p* = 0.032, n = 8 and 7) (Figure 1B). In contrast, no significant difference in locomotor velocity was detected among the three groups, indicating that motor ability in aged female mice was not impaired by either AD pathology or stroke (F (2, 21) = 1.24, *p* = 0.31) (Figure 1C). Taken together, these results demonstrate that distal MCAO aggravates cognitive impairment in Tg-2576 mice without confounding effects from motor dysfunction.

### Distal stroke exacerbates respiratory dysfunction in mice with dementia

Abnormal breathing is often observed in individuals with amyloid-related dementia,^37^ in turn, apnea exacerbates Aβ accumulation by impairing glymphatic clearance and independently contributes to the development of cognitive dysfunction.^38^ Earlier studies from our lab showed that young mice subjected to transient proximal MCAO develop stroke-induced respiratory dysfunction (SIRD), characterized by hypoventilation and apnea.^34^ To determine whether distal MCAO affects respiratory function in older Tg-2576 mice, we performed whole body plethysmography to evaluate respiratory function on day 42 post-stroke.

**Figure 2.**
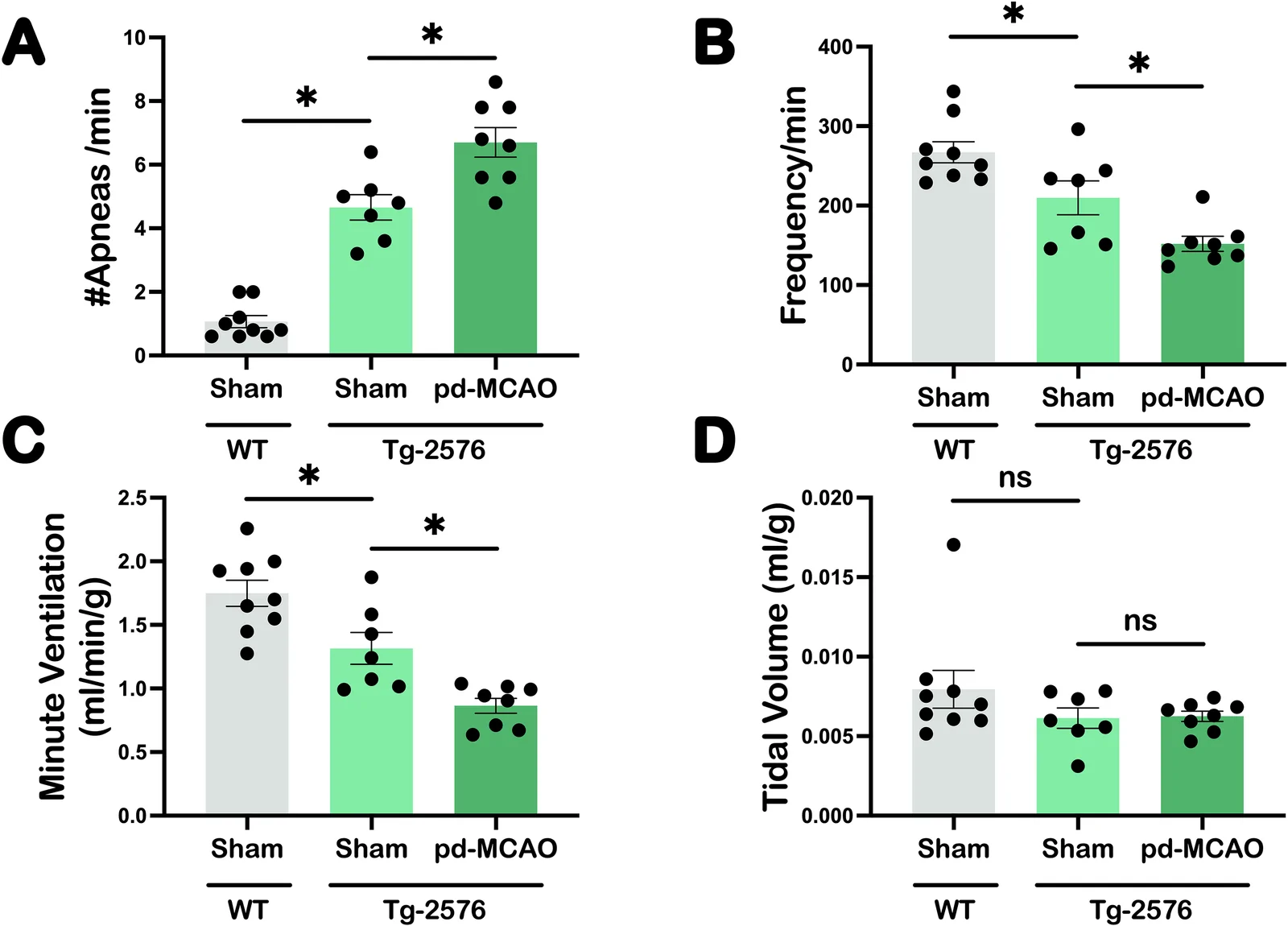
Distal stroke exacerbates respiratory disorder in Tg-2576 mice on day 42 post-stroke. (A) number of apneas per minute (F (2,21) = 68.039, p < 0.001), (B) respiratory frequency per minute (F (2,21) = 16.12, p < 0.001), (C) minute ventilation per minute/gram (MV; mL/L, per min, per g, F (2,21) = 21.74, p < 0.001 and (D) total tidal volume (TV; mL/L, F (2,21) = 1.459, p = 0.255) of WT, Tg-2576, Tg-2576-Stroke group in whole body plethysmography. *p < 0.05. ns: non-significance. n = 9 for WT, n = 7 for Tg-2576-Sham group, and n = 8 for Tg-2576-pd-MCAO group. The data are shown as the mean ± SEM. After performing the Shapiro-Wilk normality test to examine the normal distribution, One-way analysis of variance (ANOVA) was employed to analyze the data pertaining to multiple groups, subsequently, multiple comparisons were conducted using the Bonferroni test.

The whole-body plethysmography recordings indicated that WT mice exhibited regular, rhythmic breathing cycles. In contrast, Tg-2576-Sham mice showed intermittent breathing irregularities with occasional pauses. Tg-2576-Stroke mice demonstrated pronounced respiratory instability, characterized by frequent and prolonged apneic episodes, and even impairment in respiratory rhythm maintenance. Compared to WT mice, Tg-2576 mice exhibited increased apneas (Tg: 4.65 ± 0.4 versus WT: 1.06 ± 0.18, *p* < 0.001, n = 7 and 9) (Figure 2A), with suppressed respiratory frequency (Tg: 209.8 ± 21.3 versus WT: 267.07 ± 13.2, *p* = 0.039, n = 7 and 9) (Figure 2B). Tidal volume (TV; mL/g) reflects the capacity for gas exchange in a single breath, and minute ventilation (MV; mL/min per g) reflect the overall ventilation efficiency and metabolic demand. Tg-2576 mice showed no changes in TV (Tg: 0.0061 ± 0.0006 versus WT: 0.0079 ± 0.0011, *p* = 0.464, n = 7 and 9) (Figure 2D) but had a significant decrease in MV (Tg: 1.31 ± 0.12 versus 1.74 ± 0.1, *p* = 0.016, n = 7 and 9) (Figure 2C). Tg-2576 mice with stroke had a significant increase in the number of apneic events compared to sham Tg-2576 mice under room air conditions (MCAO: 6.7 ± 0.46 versus Tg: 4.65 ± 0.4, *p* = 0.002, n = 8 and 7), and stroke further decreased respiratory frequency (MCAO: 151.82 ± 9.45 versus Tg: 209.8 ± 21.3, *p* = 0.042, n = 8 and 7) and MV (MCAO: 0.86 ± 0.05 versus Tg: 1.31 ± 0.12, *p* = 0.014, n = 8 and 7) in Tg-2576 mice, while without changes in TV (MCAO: 0.0062 ± 0.0003 versus Tg: 0.0061 ± 0.0006, *p* > 0.05, n = 8 and 7). These results showed that disordered breathing is a common occurrence in Tg-2576 mice, and stroke further exacerbates these respiratory impairments.

### Distal stroke increases Aβ deposition in the cortex and hippocampus

Our behavioral and plethysmography results showed a significant change in cognitive impairment and breathing dysfunction in Tg-2576 mice which is exacerbated by stroke. Since apnea is an independent risk factor for vascular dementia,^39^ and in order to explore the underlying mechanisms of cognitive impairment as well as the changes in Aβ deposition following respiratory dysfunction, we performed immunofluorescence staining for Aβ in the cortex and hippocampus.^40^

**Figure 3.**
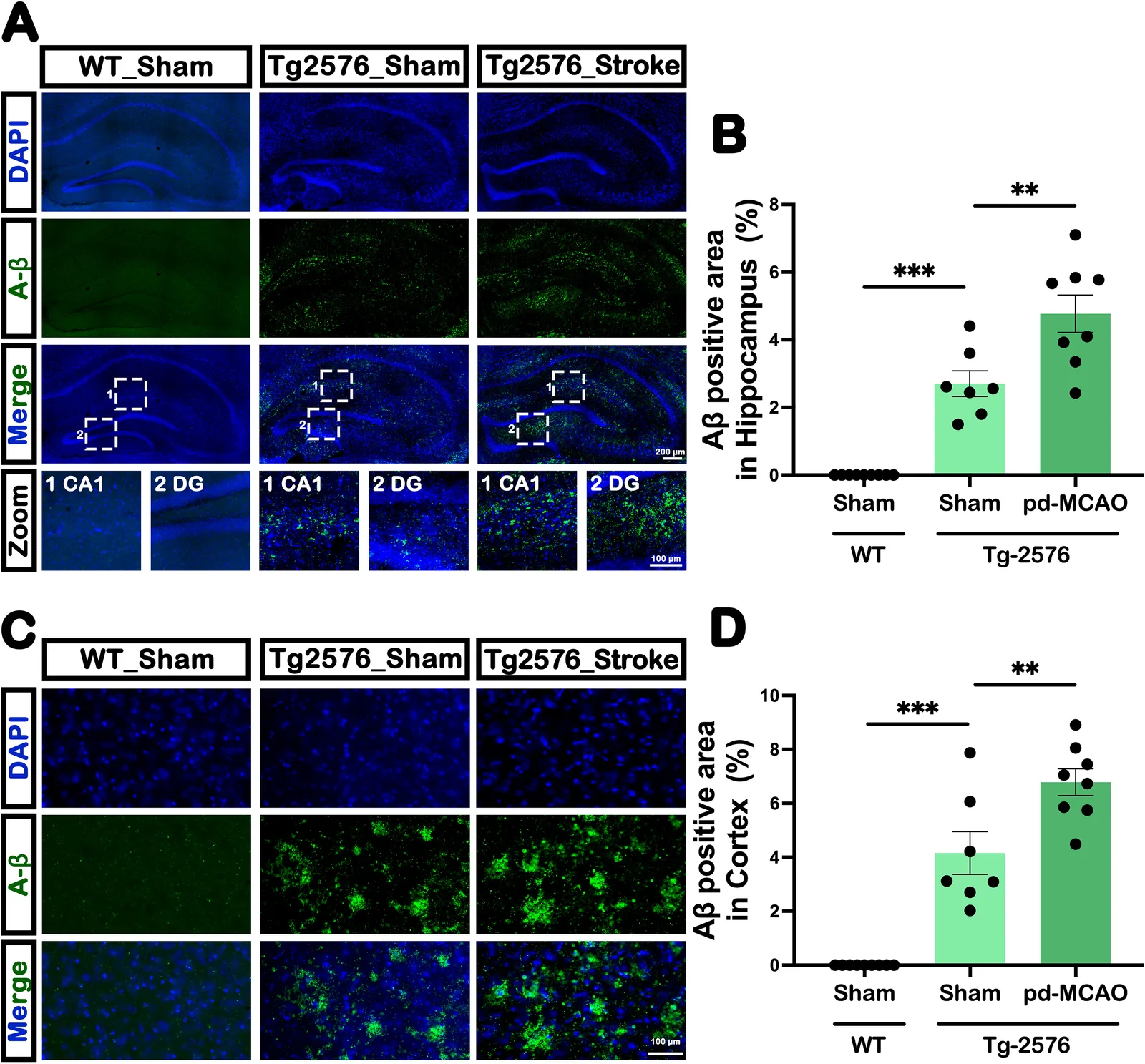
Stroke increase Aβ deposits in the cortex and hippocampus of Tg-2576 mice. (A) Representative images of the Aβ in hippocampus and (B) percentage area of Aβ in the hippocampus (F(2,21) = 44.525, p < 0.001), scale bar = 200 μm/100 μm(zoom). (C) Representative images of the Aβ in cortex and (D) percentage area of Aβ in the cortex (F (2,21) = 52.169, p < 0.001), scale bar = 100 μm. **p < 0.01; ***p < 0.001. ns: non-significance. n = 9 for WT, n = 7 for Tg-2576-Sham group, and n = 8 for Tg-2576-pd-MCAO group. The data are shown as the mean ± SEM. After performing the Shapiro-Wilk normality test to examine the normal distribution, One-way analysis of variance (ANOVA) was employed to analyze the data pertaining to multiple groups, subsequently, multiple comparisons were conducted using the Bonferroni test.

Aβ plaque burden was significantly increased in both the hippocampus (*p* < 0.001, Figure 3A,B), and the cortex (*p* < 0.001, Figure 3C,D) in the Tg-2576 group compared with the WT group, and stroke markedly upregulated the elevated Aβ plaque burden in both the hippocampus (MCAO: 4.77% ± 0.55 versus Tg: 2.7% ± 0.37, *p* = 0.003, n = 8 and 7) and the cortex (6.7% ± 0.49 versus 4.1% ± 0.79, *p* = 0.004, n = 8 and 7), as compared with the Tg-2576 sham group.

These findings suggest that stroke exacerbates both respiratory dysfunction and Aβ deposition in critical cognitive regions such as cortex and hippocampus. Therefore, we investigated the mechanisms responsible for impaired Aβ clearance. Previous studies have highlighted the essential role of the meningeal lymphatic system and cervical lymph nodes in facilitating the drainage of interstitial and cerebrospinal solutes, including Aβ.^41^ In the next phase of our study, we aimed to assess lymphatic flux function by examining LYVE1 and Aβ staining in the deep cervical lymph nodes, together with CSF Aβ levels, to further elucidate the clearance pathway disruptions contributing to Aβ accumulation and cognitive decline.

### Stroke decrease Aβ glymphatic clearance in mice with dementia

**Figure 4.**
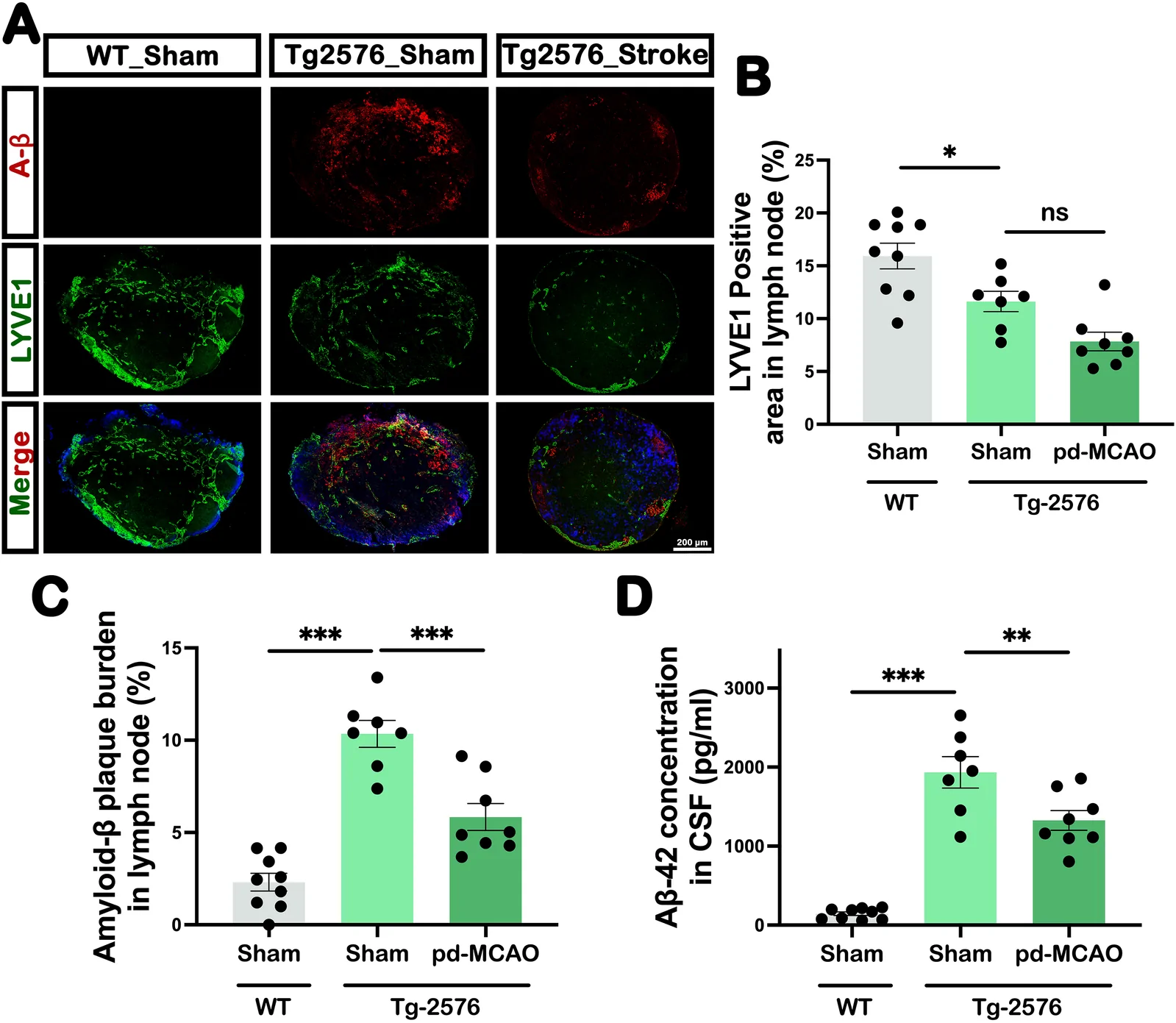
Stroke decreased the drainage function of dCLNs in Tg-2576 mice. (A) Representative images of the Aβ (red), LYVE1 (green) and DAPI (blue) in the dCLNs, (B) quantification of positive area of LYVE1 (%)(F (2,21) = 15.455, p < 0.001) and (C) positive area of Aβ plaque (%) in the dCLNs (F (2,21) = 38.729, p < 0.001). (D) Concentration of Aβ_42_ in CSF (F (2,21) = 54.557, p < 0.001). Scale bar = 200 μm. *p < 0.05; **p < 0.01; ***p < 0.001. ns: non-significance. n = 9 for WT, n = 7 for Tg-2576-Sham group, and n = 8 for Tg-2576-pd-MCAO group. The data are shown as the mean ± SEM. After performing the Shapiro-Wilk normality test to examine the normal distribution, One-way analysis of variance (ANOVA) was employed to analyze the data pertaining to multiple groups, subsequently, multiple comparisons were conducted using the Bonferroni test.

LYVE-1 and Aβ staining of the dCLNs was performed to evaluate lymphatic drainage function. Immunofluorescence results revealed a marked accumulation of Aβ (red) in the dCLNs of Tg-2576 mice (Tg:10.34% ± 0.73 versus WT: 2.3% ± 0.48, *p* < 0.001, n = 7 and 9), which was reduced in the stroke group, as compared to sham Tg-2576 controls (MCAO: 5.84% ± 0.72 versus Tg: 10.34% ± 0.73, *p* < 0.001, n = 8 and 7). In parallel, LYVE1^+^ lymphatic vessel area within the dCLNs was significantly reduced in Tg-2576 mice compared to WT (Tg: 11.62% ± 0.96 versus WT: 15.92% ± 1.21, *p* = 0.029, n = 7 and 9), indicating lymphatic structural abnormalities. Following stroke, this area showed a further decrease (MCAO: 7.84% ± 0.88 versus Tg: 11.62%±0.96) however, this reduction did not reach statistical significance (*p* = 0.072, n = 8 and 7), these data suggested compromised lymphatic function. Consistent with the amyloidogenic background of Tg-2576 mice, ELISA analysis of CSF demonstrated a significant higher Aβ_42_ level in the Tg-2576 group than in WT group (Tg: 1933.3 ± 199.25 versus WT: 143.9 ± 22.8, *p*＜0.001, n = 7 and 9). Notably, stroke significantly reduced CSF Aβ levels inTg-2576 (MCAO: 1325.4 ± 125.6 versus Tg: 1933.3 ± 199.25, *p* = 0.009, n = 8 and 7), despite increased parenchymal Aβ deposition. Together, these findings suggest that stroke reduced Aβ levels in CSF, consistent with reduced glymphatic–lymphatic export of brain-derived Aβ rather than enhanced clearance or reduced production (Figure 4).

### Stroke further exacerbates reactive astrogliosis in the RTN of mice with amyloidosis

**Figure 5.**
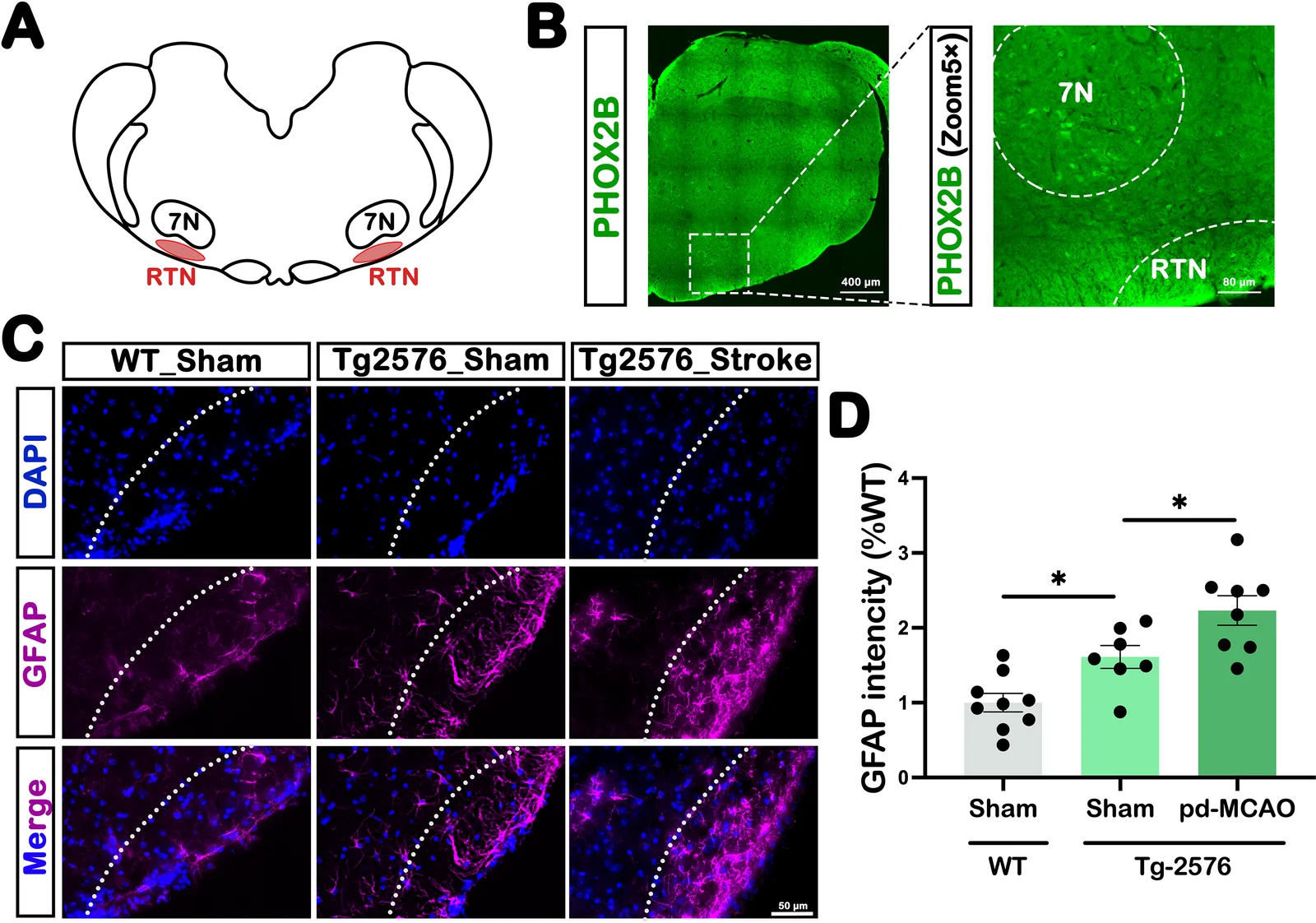
Stroke exacerbates reactive astrogliosis in the RTN of Tg-2576 mice. (A) RTN chemo-sensitive phox2B-positive neurons (area circumscribed by red solid line) are located ventromedial to the VII, medial to the pyramidal tract. (B) Histological representation of the RTN location within the brainstem, illustrating the specific area analyzed in both WT, CAA, and stroke mice, in relation to the 7th Facial Nucleus (7 N). The RTN region was identified based on Phox2B immunoreactivity and its anatomical position ventral to the 7N near the ventral medullary surface. Scale bar = 400 mm/80 μm (zoom). (C, D) Representative images show the GFAP expression in RTN from WT, Tg-2576, and stroke mice at day 42 post-ischemia (n = 9 for WT group, n = 7 for Tg-2576-Sham group and n = 8 for Tg-2576-pd-MCAO group). Scale bar = 50 µm. *p < 0.05. ns: non-significance. The data are shown as the mean ± SEM. After performing the Shapiro-Wilk normality test to examine the normal distribution, One-way analysis of variance (ANOVA) was employed to analyze the data pertaining to multiple groups, subsequently, multiple comparisons were conducted using the Bonferroni test.

To assess astrocytic activation and phenotypic shifts in the RTN region, we conducted immunofluorescence labeling for Phox2B and GFAP. For accurate regional delineation, the RTN was first defined using established anatomical cues and Phox2B immunoreactivity. Anatomically, the RTN lies in the ventral brainstem, immediately ventral to the facial motor nucleus (7N) (Figure 5A). Because Phox2B is a transcription factor enriched in RTN neurons, Phox2B staining was used as a selective marker to demarcate the RTN. Consistent with this, Phox2B-positive cells were detected in the ventral area beneath the 7N (Figure 5B), supporting identification of the RTN for subsequent quantification. GFAP immunoreactivity, a hallmark of reactive astrogliosis, was increased in Tg-2576 mice relative to WT controls and was further augmented in the Tg-2576/pd-MCAO group (Figure 5C). Specifically, GFAP expression was significantly higher in Tg-2576 mice than in WT mice (*p* < 0.05) and showed an additional increase following pd-MCAO in Tg-2576 mice (*p* < 0.05) (Figure 5D). These results indicate that stroke further enhances astrocyte reactivity in the RTN. Increased astrogliosis in this region may be associated with altered respiratory regulation and cognitive outcomes in mice with amyloidosis.

## Discussion

The present study provides several novel insights into the interaction between cerebrovascular injury, brainstem respiratory control, and waste-clearance pathways. First, stroke produces a substantial deterioration in cognitive function in AD mice, with memory performance being particularly vulnerable, supporting the notion that superimposed vascular insults can accelerate neurobehavioral decline in the context of amyloid pathology. Second, we observed marked astrocytic reactivity in the brainstem RTN, as characterized by increased gliosis-related changes. This astrocytic response in a key respiratory regulatory region was accompanied by more severe breathing abnormalities, including worsened respiratory dysfunction and a higher burden of apneic episodes following stroke in AD mice. Third, our data link respiratory impairment with compromised lymphatic drainage and reduced amyloid clearance capacity. Specifically, we observed fewer LYVE1^+^ lymphatic structures in the dCLNs, reduced Aβ clearance into the CSF, and a corresponding increase in Aβ accumulation within the cortex and hippocampus. Collectively, these findings suggest that post-stroke brainstem glial reactivity and respiratory dysregulation may be coupled to impaired clearance pathways, thereby exacerbating amyloid deposition, and contributing to the progression of AD-related pathology.

Efficient removal of Aβ from the brain is fundamental for maintaining CNS homeostasis and mitigating amyloid-related neurodegeneration, including the pathological processes underlying CAA and AD.^42,43^ For decades, Aβ disposal was largely attributed to cellular and local mechanisms, such as phagocytosis and autophagy, or to passive movement of soluble waste into venous pathways.^44^ More recently, converging clinical observations and experimental studies have shifted this perspective by highlighting the glymphatic pathway and meningeal lymphatic vessels as major routes for brain-wide fluid and solute clearance.^45,46^ In this updated model, CSF enters the brain along periarterial spaces and is propelled through perivascular channels, enabling convective exchange with interstitial fluid (ISF) and promoting the transport of solutes, including Aβ, within the parenchyma.^47^ Waste-laden ISF subsequently drains along perivenous routes toward the subarachnoid space, where it interfaces with meningeal lymphatic outflow and ultimately reaches the systemic circulation.^48,49^ Importantly, this anatomical continuum provides a direct conduit for CSF-derived solutes to access the dCLNs, thereby coupling CNS waste removal to peripheral immune surveillance and downstream clearance mechanisms.

In this context, disruption of glymphatic–lymphatic transport is expected to limit Aβ efflux, promote parenchymal retention, and potentially intensify subsequent neuroinflammatory and behavioral deficits.^26^ Indeed, impaired glymphatic flow has been associated with reduced interstitial solute clearance and increased accumulation of Aβ, changes that can facilitate plaque formation and accelerate amyloid-related pathology.^50^ Importantly, glymphatic function is also sensitive to breathing dynamics, respiratory instability, particularly apnea-like pauses, has been reported to reduce glymphatic exchange efficiency.^51,52^ In line with this concept, our prior work showed that aged CAA dementia mice exhibit abnormal breathing patterns and cognitive impairment accompanied by prominent astrocytic reactivity in the brainstem RTN.^20,53,54^ Building on these findings, we investigated whether an ischemic insult in AD mice would further compromise RTN-mediated respiratory control and, in turn, disrupt glymphatic clearance, thereby promoting greater Aβ accumulation, consistent with our current observations linking post-stroke respiratory dysfunction to impaired lymphatic drainage and increased Aβ burden.

First, we found that ischemic stroke in AD mice further aggravated both breathing abnormalities and cognitive deficits. Consistent with our prior work in stroke models, cerebral ischemia can precipitate respiratory disturbances and episodes of intermittent hypoxia in mice.^34^ Such intermittent hypoxia may promote amyloid accumulation through several non-mutually exclusive pathways. Respiratory instability can fragment sleep architecture, which is expected to compromise glymphatic-dependent clearance of solutes from the brain and CSF.^55^ In parallel, recurrent intermittent hypoxia has been reported to increase amyloidogenic processing and Aβ production, potentially accelerating amyloid burden and worsening learning and memory performance in AD mice.^56,57^ These mechanistic links will be examined in our further studies.

The distal MCAO stroke model produces cortical infarcts without directly injuring brainstem neurons.^34^ However, it triggers astrocytic activation in remote brainstem regions, such as the RTN. Our previous work suggested that this is mediated by enhanced TGF-β signaling,^20^ where microglia-derived TGF-β diffuses globally, activating Smad2/Smad3 pathways in astrocytes and driving chondroitin sulfate proteoglycan (CSPG) overexpression, a key component of glial scarring.^58^ After ischemic stroke, astrocytes undergo reactive changes and display substantial heterogeneity across brain regions and disease contexts.^59^ Depending on the local milieu, reactive astrogliosis can encompass inflammatory-like programs as well as repair-associated responses, and prior work has used molecular signatures to distinguish these functional states.^60,61^ Reactive astrogliosis is also closely linked to extracellular matrix remodeling, including increased production of CSPGs and glial scar formation. Experimental studies suggest that dampening maladaptive reactive astrocyte signaling can reduce scar-associated features in vitro and in vivo, supporting the therapeutic rationale for targeting excessive astrogliosis after ischemic injury.^62^ In addition, clinical observations have implicated upstream pathways such as TGF-β signaling in shaping reactive astrocyte responses in dementia-related conditions,^63^ raising the possibility that similar signaling may contribute to RTN astrogliosis after stroke.

We characterized Aβ burden and regional distribution using immunofluorescence staining and ELISA. In AD mice, similar to what is reported in AD patients, Aβ pathology developed early and increased with age. Prominent thioflavin-S–positive fibrillar Aβ was evident in regions such as the thalamus, inferior colliculus, and hippocampus, whereas more diffuse parenchymal Aβ deposition was broadly detected in the cortex.^64,65^ In line with these established deposition patterns, our immunofluorescence analyses further showed that ischemic stroke markedly increased Aβ accumulation in both the hippocampus and cortex. Together, these findings support the conclusion that stroke accelerates amyloid pathology within brain areas critical for learning and memory. This increase in Aβ burden may reflect, at least in part, reduced clearance efficiency secondary to post-stroke breathing abnormalities, including altered respiratory rhythm and apnea-like events, which are tightly coupled to perivascular fluid transport and drainage. The preferential exacerbation of amyloid pathology in the hippocampus and cortex further suggests that these cognition-related regions are especially susceptible to the combined impact of vascular amyloidopathy and ischemic injury, a convergence that may hasten cognitive deterioration in the setting of comorbid AD and stroke.

Notably, although stroke led to a marked increase in Aβ accumulation in the hippocampus and cortex, we detected significantly lower Aβ levels in the CSF and deep cervical lymphatic compartments. This opposite trend is consistent with reduced glymphatic–lymphatic export of Aβ in AD mice after ischemic injury. Under physiological conditions, glymphatic transport supports the movement of interstitial solutes, including Aβ, from the parenchyma into CSF and subsequently toward peripheral lymphatic drainage sites such as the deep cervical lymph nodes.^66^ Following stroke, this clearance route may be compromised, potentially due to disruption of respiratory-coupled CSF dynamics that normally help drive perivascular flow. As a result, diminished Aβ efflux would be expected to favor retention and further buildup within cognition-relevant regions, thereby accelerating amyloid pathology and functional decline. Together, these data emphasize the importance of glymphatic–lymphatic drainage for brain homeostasis and suggest that this pathway is particularly vulnerable when AD is compounded by ischemic stroke.

While our data support an association between RTN astrogliosis and reduced glymphatic clearance, the causal pathway remains to be established. Dysfunction within the RTN could plausibly destabilize respiratory control—manifesting as more frequent apnea-like events, hypoventilation, and irregular breathing patterns.^67^ Such disturbances may secondarily influence cardiopulmonary coupling and systemic venous return, producing abnormal intracranial pressure (ICP) dynamics and irregular pressure oscillations.^68^ Because glymphatic transport depends in part on vascular pulsatility and pressure gradients that help propel CSF through perivascular spaces, attenuated or erratic respiratory-linked ICP modulation could compromise CSF movement and reduce clearance efficiency. Direct assessment of ICP fluctuations alongside measures of RTN activity and breathing would help clarify whether this respiratory–pressure axis mechanistically connects RTN pathology to impaired glymphatic function.

Several limitations of the present study should be acknowledged. The experiments were performed only in female mice, which may restrict the generalizability of the conclusions. Sex hormones are known to modulate cerebrovascular physiology, respiratory control neuroinflammatory responses and glymphatic clearance efficiency.^69,70^ Given these sex-dependent influences, combining males and females within the same cohorts without sufficient power and stratification could introduce biological variability and confound interpretation; accordingly, we focused on a single sex in this initial study. Female mice were selected because this murine model shows faster amyloid accumulation in females.^31,32,71^ Consistent trends have been reported clinically, where females with CAA or AD often present with greater disease burden and cognitive impairment.^72^ Future work should include both sexes with appropriately powered, sex-stratified designs to define sex-dependent trajectories of AD progression and to determine how ischemic stroke differentially impacts pathology and functional outcomes.

In addition, distal MCAO is an acute focal ischemic injury model and does not fully recapitulate the chronic and heterogeneous vascular pathology observed in AD patients, including small vessel disease, cerebral hypoperfusion, microinfarcts, and vascular dysfunction. However, compared with the intraluminal filament MCAO model, distal MCAO generally produces a more localized cortical infarct and less extensive ischemic injury. Thus, in the context of Tg2576 mice with existed AD like pathology, this model may reflect a clinically relevant scenario in which a focal ischemic event is superimposed on an AD-related pathological background. Moreover, the absence of a WT-pd-MCAO group in the present study limits our ability to distinguish the independent effects of stroke from those associated with the AD-related background. Future studies incorporating both WT and Tg2576 mice with sham and pd-MCAO conditions will permit two-way ANOVA to evaluate the main effects of genotype and stroke, as well as their interaction. Studies using chronic hypoperfusion and small-vessel disease models will also be needed to further characterize the vascular contributions to AD progression.

Another limitation of the present study is that astrocyte reactivity in the RTN was assessed only at a single late time point (42 days post-stroke), which does not capture the temporal evolution of reactive changes. Because post-ischemic astrogliosis is dynamic and stage-dependent, future studies should profile RTN astrogliosis longitudinally across multiple time points (e.g., 7, 14, 28, and 42 days) and determine how these changes relate to respiratory control and cognitive outcomes.

Moreover, glymphatic dysfunction in the current study was deduced indirectly from an inverse distribution pattern, reduced Aβ levels in the CSF and deep cervical lymphatic compartments together with increased parenchymal deposition, rather than from direct measurements of fluid transport. Future studies should incorporate approaches that quantify glymphatic flow more directly, such as intrathecal tracer infusion with ex vivo mapping, two-photon imaging to visualize perivascular tracer movement, or in vivo imaging modalities (e.g., MRI using gadolinium-based tracers). Incorporating these methods would provide stronger mechanistic evidence linking post-stroke respiratory dysregulation and RTN astrocytic reactivity to reduced glymphatic–lymphatic clearance.

In conclusion, our findings support a previously unrecognized connection between ischemic stroke and reduced glymphatic–lymphatic clearance in AD mice, accompanied by increased Aβ accumulation in the brain. The data further indicate that post-stroke respiratory abnormalities, together with prominent astrocytic reactivity within the brainstem RTN, are closely associated with disruption of waste-removal pathways. Collectively, these results emphasize the importance of preserving glymphatic function in cerebrovascular disease and suggest that interventions aimed at modulating maladaptive astrogliosis and stabilizing respiratory control may represent promising therapeutic directions to mitigate amyloid pathology and functional decline.
